# Improving Wellness for LGB Collegiate Student-Athletes Through Sports Medicine: A Narrative Review

**DOI:** 10.1186/s40798-018-0163-y

**Published:** 2018-11-06

**Authors:** Mikalyn T. DeFoor, Lara M. Stepleman, Paul C. Mann

**Affiliations:** 1 School of Medicine, Medical College of Georgia, Augusta University, Augusta, GA 30912 USA; 2 Department of Psychiatry and Health Behavior, Medical College of Georgia, Augusta University, Augusta, GA 30912 USA; 3 Department of Pediatrics, Medical College of Georgia, Augusta University, Augusta, GA 30912 USA; 4 Center for Bioethics and Health Policy, Medical College of Georgia, Augusta University, Augusta, GA 30912 USA

**Keywords:** Lesbian, gay and bisexual, LGB, Student-athletes, College, Sport, Sexual minorities, Barriers, Sports medicine, Youth

## Abstract

In comparison to their heterosexual peers, lesbian, gay, and bisexual (LGB) student-athletes encounter substantial challenges during their intercollegiate and professional athletic careers including detrimental stereotypes, harassment, and discrimination. Such non-inclusive environments promoted throughout the current Western culture of sport are notably associated with higher incidences of mental health and substance use disorders among LGB athletes across youth, collegiate, and professional sports. There have been significant gains at the collegiate level to address LGB-inclusive practices aimed towards administrators, educators, coaches, and student-athletes; however, there is currently no literature that addresses the unique role of the sports medicine team. As first-line healthcare providers for student-athletes, sports medicine physicians and athletic trainers are uniquely positioned to support collegiate LGB athletes through affirming sexual identity, recognizing distinctive health risks, and advocating inclusivity within the athletic training room. By examining major themes of concern among current LGB student-athlete experiences across the unique setting of US colleges and universities, this review article aims to further identify opportunities for sports medicine providers to promote positive health outcomes and improve the overall wellness of collegiate LGB student-athletes.

## Key Points

- For lesbian, gay, and bisexual (LGB) student-athletes, the collegiate environment is a particularly vulnerable transition magnified by concerns of peer rejection, “outing” sexual orientation, and hypermasculine societal norms, further complicated by a lack of social support and institutional inclusive policies within the current heteronormative culture of American sport.
- Majority of LGB youth perceive sport participation and athletic environments as unsafe and non-inclusive and, therefore, do not benefit from the widely reported physical and psychosocial health benefits of athletic participation and team sport.
- Sports medicine providers have the unique opportunity and professional responsibility to provide a visible presence of support and inclusion, while recognizing perceived barriers to care in order to improve the overall wellness of LGB student-athletes through multidisciplinary care.

## Background

Many professional athletes have been celebrated in the media for “coming out” with their sexual identities in recent years which has seemingly fostered increased visibility, acceptance, and awareness for lesbian, gay, and bisexual (LGB) athletes across American sport culture. Some prominent examples include Jason Collins who received significant media attention for coming out as the first openly gay National Basketball Association (NBA) player after retiring in 2013 [1, 2], Brittney Griner who came out as lesbian after being the first pick in the 2013 Women’s National Basketball Association (WNBA) draft [1, 3], and Michael Sam who became the first openly gay prospect entering the National Football League (NFL) draft in 2014 [1, 2]. These notable validations, while a significant gain in the positive direction for LGB awareness, may send a false perception that the Western culture of collegiate sports has also become more open and accepting of gay and lesbian athletes. As yet, though, there has been no reported correlation between gains experienced by professional athletes with a more welcoming and inclusive experience for younger lesbian, gay, and bisexual athletes across US colleges and institutions [4].

Although research has shown significant progress in inclusivity and acceptance of the LGB community across college campuses over the years [5, 6], the unique experience of a sexual minority identity within intercollegiate athletics is under-studied and under-reported. The National Collegiate Athletic Association (NCAA) has made significant strides in developing inclusivity policies for sexual minority student-athletes and best practices specifically designed for athletic administrators, coaches, and student-athletes [6, 7]. However, no similar “best practice” guidelines address the pivotal role of sports medicine teams in creating a healthy, inclusive, and welcoming environment. Additionally, there is a paucity of research addressing how sports medicine providers can better address the crucial health disparities of LBG athletes such as higher incidences of substance abuse [8, 9] and disordered eating [10].

Despite opportunities for sports medicine providers to positively impact the health of LGB student-athletes, there has been a lack of research highlighting health engagement in sports through combatting the discrepancies among physical exercise participation, substance abuse and mental health disorders, non-inclusive environments, stereotypes, and discrimination among LGB adolescents when compared to their heterosexual peers. This article explores current biases and barriers that collegiate sexual minority student-athletes encounter with a goal towards better defining opportunities for the sports medicine team to holistically promote overall wellness by meeting the needs of LGB student-athletes within the athletic training room. Through investigation of the research literature available, we summarize major themes of concern among LGB student-athletes and identify opportunities for future research and institution educational policies necessary to improve safety and inclusivity for intercollegiate LGB athletes.

## Methods

The findings of this review will be of primary interest to the sports medicine healthcare providers (athletic trainers, physical therapists, and clinicians) who regularly participate in the preventative health and injury management of all student-athletes throughout US colleges and universities. Relevant studies were identified utilizing electronic databases of Google Scholar, Galileo, Web of Science, SPORTDiscus, and PubMed using combinations of keywords that included “LGBT,” “sexual minorities,” “sports medicine,” “sport,” “athletes,” “barriers,” “youth,” “college,” and “homophobia.” Forward citation and reference lists of selected articles were examined to identify additional articles of interest. A review of current NCAA Publications was also conducted for relevant inclusion policies for LGB student-athletes and sports medicine guidelines. Only papers written in English and published with a focus on youth, collegiate, and professional athletes across the USA were considered for this review. A systematic review of the literature was intentionally not conducted for the purposes of this article because, instead of an exhaustive review with narrowed selection criteria, our focus is to identify major overall themes in the literature of concern expressed among LGB student-athletes to be considered in future research, health, athletic, and academic agendas.

While acknowledging the importance of advancing transgender health and inclusion in collegiate sports, the scope of this paper is principally focused on the experiences and health needs of lesbian, gay, and bisexual athletes. We use the term LGB throughout this paper except for where this differs from the terminology used to describe samples from research studies summarized herein. In these instances, the original terminology used by the study authors is maintained, such as lesbian, gay, bisexual, and transgender (LGBT) or lesbian, gay, bisexual, transgender, and queer (LGBTQ).

## Major Themes of Concern

### Historical Concerns of LGB Athletes

In contrast to professional athletic endeavors, educational institutions and universities provide extracurricular athletics to enhance their scholarly mission with a goal towards more comprehensively advancing the academic, social, emotional, and physical development of student-athletes. Athletic opportunities augment academic attainment, teaching life skills, broader perspectives, and positive character development while furthering self-knowledge, self-esteem, and citizenship through the concept of diversity and competition [11]. It is well-accepted that participation in athletics and organized team sport is positive for fostering peer relationships, inclusivity, and self-confidence and even boosting academic performance [12–14], but does this also hold true for sports participation by the LGB student-athlete?

Historically, athletics and organized team sports have been specifically of concern for LGB students. Despite the vast diversity of ethnicity, socioeconomic status, geographic background, and even sexual orientation, heterosexist and homophobic attitudes primarily dominate the world of sport [6, 15]. In the *2012 LGBTQ National College Athlete Report* that surveyed 8,481 student-athletes across 164 NCAA institutions, 394 individuals (5%) self-identified as LGBTQ [16]. This national study revealed that one in four LGBTQ student-athletes reported pressure to be silent about their sexual orientation among teammates and within the culture of college sports. These athletes were also more likely than heterosexual athletes to have other marginalized identities including female gender, racial or ethnic minority, reported disability, or non-Christian religious beliefs.

The focus of rules and regulations has historically fixated on the eligibility of transgender athletes in the professional setting [11, 17, 18]. In 2012, the NCAA association-wide Committee on Women’s Athletics and Minority Opportunities and Interests Committee first published guidelines regarding inclusion policies of LGBT student-athletes and staff [7]. This resource made a tremendous effort to emphasize and define best practices and responsibilities of coaches, administrators, and student-athletes in creating a non-discriminatory environment of inclusivity. However, those guidelines did not address the unique role of the sports medicine team that provides regular and routine medical care to participating student-athletes.

Under the guidance of a designated medical director, sports medicine providers have obligations to ensure equitable access to quality medical care for all participating student-athletes for the prevention, diagnosis, and management of injuries as well as return to play decisions [19]. As highlighted in the *NCAA Sports Medicine Handbook*, revised in July 2014, safe competition environments and equitable medical care without “discrimination on the basis of sexual orientation” are key elements for student-athlete injury prevention [20]. As the athletic training room serves as a buffer zone for injured athletes, the sports medicine team has the unique opportunity to play a pivotal role in transforming current non-inclusive sport culture and heterosexist norms by affirming all student-athletes, challenging homophobic attitudes, and specifically advocating for the health needs of LGB student-athletes [15].

### Vulnerability of the Collegiate Setting

The transition to the intercollegiate environment for any student-athlete can be one of the most socially vulnerable times influenced by pressures to maintain elite athletic status, meet expectations of societal norms, and maintain their athletic identity [21, 22]. However, it can also be one of the best stages to nurture a sense of personal belonging and identity, promote the value of teamwork and work ethic, and teach the ability to overcome personal differences and disagreements to accomplish the greater goal of the team [23, 24]. Collegiate athletes are particularly vulnerable during their first-year transition to college due to the unique attributes of the college environment. Major concerns for both athlete and non-athlete first-year students include the need to budget time and money, responsibility for student loan debt, negotiating ongoing peer pressure, and exploring sexuality [25, 26].

There are conflicting reports regarding different responses to stressful events experienced between collegiate athletes and non-athlete collegiate peers, specifically in the context of private versus public institutions [26, 27]. One study from the perspective of a single Division I private institution identified relationship conflicts, multiple responsibilities, lack of sleep, and time demands from extracurricular activities to be statistically more significant underlying stressors complicating their first year of college when compared to non-athlete peers [25]. A more recent study additionally reported student loan burden and financial stress as a significant contributing factor to an increased likelihood of discontinuing college education [28]. Like non-athletic participating peers, collegiate athletes are faced with the challenge of being away from home and major support systems for the very first time, yet still being financially dependent on their families and/or student loans [27].

For the first-year student-athlete, stressors may be potentiated during the initial transition to college by fear of injuries, not securing playing time, and failing to maintain elite athletic status while transitioning to a higher level of play [26]. Additionally, first-year student-athletes are suddenly faced with challenges of time management and missing class for team travel, resulting in less time and opportunity to acclimate to college life. It is easy to imagine that these very really stressors to compete athletically at a high level are magnified in the experience of an LGB student-athlete with the additional pressure to perform and conform in the largely heteronormative culture of sport.

For LGB student-athletes, social support and feelings of acceptance are magnified areas of vulnerability when transitioning to a college environment as they are also faced with the challenge of coping with sexual orientation stigma [29, 30]. Studies suggest that LGB youth are particularly more reliant on peer social support for their emotional well-being and perceived sexuality acceptance, especially when perceived family support is low and fear of parental rejection is present [31–33]. After interviewing 10 former gay or lesbian college student-athletes from NCAA and National Association of Intercollegiate Athletics (NAIA) institutions, Barbour and colleagues reported that some athletes were explicitly advised against publicly sharing their sexual orientation, while others described pressure by the student code of conduct, discriminatory behavior from peers, or limited playing time indicating that their sexual orientation was not acceptable [34]. With clear signs of discrimination and harassment, many athletes reported feeling the need to be cautious of revealing their true sexual identity for fear of negative consequences or becoming a distraction to the team environment.

Additional studies assessing LGBT ally attitudes portrayed by heterosexual student-athletes identified 35% of respondents being neutral to the need for increased LGBT-inclusive policies and almost 20% being opposed to more inclusive policies in intercollegiate athletics [35]. Female gender, liberal political ideology, and not knowing an LGBT athlete are all factors associated with increased support for LGBT-inclusive policy among heterosexual student-athletes. Increased perception of team acceptance of LGBT individuals and increased frequency of experiencing homophobic language in the team setting are also significantly correlated with greater support for LGBT protective guidelines among allies [35, 36].

### Non-inclusive Heteronormative Environment

Participation in team sport is a major avenue for socialization and peer acceptance. However, Western athletic culture reinforces the perception of male-dominated, heterosexist participation bounded by conventional conceptualizations of athleticism, masculinity, and femininity which significantly influence athletic participation and self-perception of LGB student-athletes [37, 38]. Jones and colleagues reported that lack of an inclusive and welcoming environment was found to be the primary barrier to participation in sport-related physical activity for LGBT individuals [39]. A perceived lack of both social support and peer acceptance promotes feelings of isolation and leads to poor athletic and academic performance among LGB student-athletes. This constellation of social isolation and poor performance can further spiral into doping, athlete drop-out, or a multitude of psychosomatic illnesses, including eating disorders, anxiety, depression, substance abuse, and self-harm, all of which are associated with increased risk for suicide contemplation [40]. The health of an individual is likely to suffer when provider homonegative attitudes are perceived, and subsequently, injured or ill LGB athletes may delay treatment or may be less likely to seek regular care [15]. Potential consequences of delayed care include preventable accidental and recurrent injuries, poor response to treatment, and longer periods before return to play, as well as prolonged illnesses such as malnutrition, dehydration, mental health issues, and psychological disorders [40].

Barber and Kane reported that the single greatest barrier to participation among LGB youth in physical education and athletics is the near universal silence regarding sexual orientation and gender identity, often referred to as the “elephant in the locker room” [4]. Sexual minority athletes reported feeling as if their acceptance within their team sport is contingent on their athletic performance and as if they must use their athletic skill to mediate the stigma associated with their sexual orientation [41]. It is also historically supported that rates of peer rejection are the best predictor of youth delinquency, drop-out rate, and adult maladjustment [42]. Not surprisingly, these negative experiences as a result of a non-inclusive climate negatively impact not only athlete self-identity, but also measures of educational outcomes, personal development, and social interactions [40, 43].

While it is evident that traditional locker room culture poses a significant barrier factored into an LGB athlete’s experience with competitive sport [4], this perceived challenge in relation to sport participation is exacerbated by discomfort in the changing room and pressure to adapt heterosexual norms [44]. Osborne and Wagner reported a study of over 1,000 students in a Pennsylvania high school in which males involved in competitive team sports were three times more likely to express homophobic beliefs than non-participating peers [45]. Gill and colleagues conducted a study in which all students admitted to recognizing the occurrence of homophobic name calling and harassment, yet low rates of action and lack of intervention sent a message that such action is tolerated and met without reprimand [46]. Despite an increase in awareness of homophobia over more recent years, few administrators, educators, or students openly and publicly challenge heteronormative behaviors. Whether this is due to fear of peer scrutiny or fear of one’s own social acceptance, Morrow and Gill report disconnect between the number of educators that acknowledge this dilemma and believe they offer a safe space versus the number of LGB students who can identify a perceived safe space in the school and athletic environments [47].

### In-house Harassment and Discrimination

Researchers confirm that LGB athletes across the continuum are at the highest risk of harassment and abuse, with psychological maltreatment being the center of non-accidental violence [40]. Not surprisingly, this harassment and abuse arise from prejudices that are expressed through power differentials. LGB collegiate athletes are oftentimes reluctant to reveal their sexual identity and feel compelled to adopt identity negotiation and impression management tactics to avoid prejudice and discrimination [48]. A study of five NCAA Division I campuses that analyzed how athletic teams perceive and respond to teammates of diverse backgrounds found that orientation elicited the strongest negative responses [6]. From this study, it was concluded that hostility towards LGBT teammates likely exists in nearly all sport teams across the nation.

In a 2011 survey, 21% of LGBT student-athletes reported receiving derogatory remarks through social media and other electronic means, almost double that for their heterosexual teammates on campus [16]. Homophobia can be expressed at the level of an individual athlete through derogatory language, homophobic attitudes, hostile team environment, and discrimination by limited playing time. Furthermore, homophobic attitudes promoted at the level of an athletic institution can be expressed through scholarship conditions, career length, lack of inclusive policy, and inequality and disrespect in sporting environments [40]. Balancing the specific interest of LGBT student-athletes to participate in athletics with the interest of institutions to ensure safe and equitable experiences for all students to compete against others of a similar skill level has been a voiced area of concern for administration [11]. The NCAA currently provides a nonbinding position statement to its member institutions and universities governing participation by LGBT intercollegiate athletes in competition under its scholarship [11, 18].

### Male and Female Student-Athlete Stereotypes

Gender, sexuality, and sport are complexly intertwined disciplines that bring about a vast array of perceived stereotypes and discrimination concerning male and female LGB student-athletes. Furthermore, in athletic participation, masculinity and femininity are often conflated with sexual orientation. Televised sports in the media consistently provide a narrow and restrictive portrait of masculinity which further reinforces the Western culture of heterosexual norms. Sport for male athletes is intertwined with traditional notions of masculinity and heterosexuality, whereas sport for women disrupts typical gender constructs [46]. Studies show that female athletes historically enter a paradoxical environment by entering a culture of inherent and expected masculinity [49]. Lesbian female student-athletes are often expected to have superior athleticism to their heterosexual teammates, which is often qualified as their ability to “play like a man” [50]. On the other hand, gay male student-athletes are presumed to have talent inferior to their heterosexual teammates due to an assumed substandard display of masculinity and physical strength [51]. In a retrospective study of ten adolescents (age 19 to 22 years) self-identifying as lesbian or gay across the southeast US, lesbians perceived sport participation as a venue to explore their sexual identity to a similar extent that gay men rejected sport participation [52]. This study suggests that sport provides a context for developing personal rather than social identity, and the effects of prejudice based on sexual identity diminish participation and access to sport.

Sports reporter Karen Crouse summarizes the media attention of female athletes as two extremes—“the sum total of her physical assets–or invisible” [53]. Perception as a feminine and attractive woman equates to acceptance yet sexual objectification in the world of sports, whereas perception as too masculine equates social deviance and lesbianism [54, 55]. This contradiction between femininity and athleticism leads to a position of social invisibility and belittles the role of the female athlete in sport participation [56]. Contrary to the common perception that sexual minority females are more athletic than heterosexual peers and overrepresented in sport, LGBT females engage in less moderate to vigorous physical activity and team sports than heterosexual peers in early adolescence [57]. Additionally, sport climates are often worse for lesbians who are also racial minorities, as they face a unique challenge of intersectional discrimination based on race, sexual orientation, and gender. Walker and Melton explored this sexual prejudice of minority lesbians in intercollegiate sports and reported that all participants felt that it was typical to hide sexuality when working in intercollegiate sports, most likely because the white, heterosexual, and male-dominated administration would not be accepting [58]. Many believed that refusing to conform to traditional gender norms would drastically reduce career opportunities, so instead they reported managing expression of their sexual orientation in the workplace to be more of a barrier than their racial identity.

Previous studies suggested that even as young adolescents involved in sport, males are often placed in highly gender-segregated environments reassuring stereotypical hypermasculinity and further socializing athletes to undermine femininity and LGBT individuals [59]. Furthermore, many collegiate sporting activities take place in close proximity situations involving direct male-on-male physical interaction in contact sports, locker rooms, showers, and recovery whirlpools, and evidence finds that men use this time to reinforce their heteromasculinity through male bonding [60]. Studies of “locker room talk” over the past three decades repeatedly suggest an environment that reinforces hegemonic masculinity, revealing dialogue that traditionally objectified women, promoted sexist attitudes, and resulted in gay-bashing [1, 2, 61, 62]. Worthen reported a significant relationship in the collegiate environment between males participating in athletics and the Greek letter fraternity system and negative attitudes towards LGBT individuals [60]. The study showed that male students who support more traditional gender roles are also more likely to be homophobic, thus supporting the belief that athletics and Greek membership traditionally endorse anti-LGBT attitudes. Murnen and Kohlman additionally showed that male athletes and fraternity members have higher levels of hypermasculinity than non-participating males, suggesting that the development of heteromasculinity among college men may likely be reified though strict cultural norms that reinforce homophobic attitudes [63].

### Prevalence of Mental Health Issues and Substance Use

Discriminatory practices at the individual or institutional level are identified as risk factors for depression, social isolation, and hopelessness and in turn increase the risk for contemplation of suicide. Over 30 years of research underscores the disproportionately higher rates of depressive symptoms, substance abuse, suicidal ideation, and suicide attempts among LGBT youth [6]. Additionally, sexual minority adolescents generally report lower overall self-esteem which could be a significant barrier to physical activity participation among peers [64]. This barrier to physical activity due to negative self-image continues to create a vicious cycle of negative self-esteem, physical inactivity, and poor academic performance further burdened by social isolation and peer rejection [57].

Veliz and colleagues performed a national study that reported sexual minority athletes to be at greater risk of substance use suggested by their marginalized status within the context of sport [9]. Although all collegiate athletes were more likely to binge drink when compared to non-athlete peers, sexual minority collegiate athletes were reported at an additional greater risk of using cigarettes, alcohol, and/or marijuana in the past 30 days compared to heterosexual collegiate athletes. Despite athletic participation being historically supported as a protective measure among collegiate athletes with respect to cigarette and marijuana use, this positive trend was not present among sexual minority collegiate athletes [65, 66]. Sexual minority collegiate athletes also indicated higher odds of being diagnosed or treated for a substance use disorder during the past 12 months when compared to heterosexual athletes and non-athletes but had similar odds when compared to sexual minority non-athlete peers [64]. A possible explanation may include difficulty of sexual minority athletes to maintain an athletic identity within a social environment that traditionally fosters homophobic norms.

### Making Sport Safer for Future Generation LGB Youth

If LGB youth do not perceive sport and physical activity environments as safe, they will be less likely to participate, sacrificing the academic improvements and wellness associated with sport participation and physical activity [67]. With the obesity epidemic and overall lack of physical activity consuming the health of our nation, it becomes even more essential to promote sport-related physical activity as a means for improved health outcomes in our nation’s youth, include the marginalized LGB population [68]. Calzo and colleagues reported sexual minority youth were 46–72% less likely to participate in team sport each week than their heterosexual peers [57]. An additional study reported that sexual minority adolescents age 12–18 years reported 1.2–2.6 h per week less of moderate to vigorous activity than heterosexual counterparts [69].

The *2015 National School Climate Survey* of over 10,500 students between the ages of 13 and 21 from over 3,000 unique school districts reported that over one third of LGBTQ students avoided gender-segregated spaces in school, with avoidance of the locker room (37.9%) and physical education/gym class (35.0%) only ranking slightly behind bathrooms (39.4%) as the number one avoided space in the school setting [70]. In addition, most LGBTQ students reported avoiding extracurricular school activities (65.7%), and about a quarter avoided extracurricular activities often or frequently (23.0%) because they felt unsafe or uncomfortable. Just over one in ten students (11.0%) reported school athletics as being one of the most commonly perceived forms of gender segregation in school activities. These data suggest that despite efforts to increase attention and awareness of sexual minority youth over recent years, LGB youth continue to be overlooked in practices of inclusivity, which can discourage participation in extracurricular school activities.

## What We Have Learned: A Need for Sports Medicine Culture Change

The overwhelming team benefits of challenging the current homophobic culture of sport have been well-described to include improvements in team performance, team chemistry, and learning environments [4]. By removing barriers to athletic participation and enhancing sport safety in the intercollegiate setting, future generations of LGB youth will be more likely to benefit from increased physical activity and healthy lifestyle habits [5]. While the NCAA’s current lack of a firm position does not publicly dissuade LGB athletes from competing at the collegiate level, the NCAA misses the opportunity to take a stance and openly advocate for an inclusive and harassment-free environment for LGB student-athletes through formal policy regulations and guidelines. Although the greatest change still needs to occur within governing groups over intercollegiate sports and institutions of education, at the individual level, there remains the perception that a single individual cannot produce a meaningful change in the sport climate [4]. However, individual providers within the intimate atmosphere of the athletic training room can have a large impact on treating the whole student-athlete and broadening the scope of inclusive, multidisciplinary care.

Research supports that the LGBT student who is able to identify a supportive teacher or staff member displays better academic performance than the LGBT student-athlete who is unable to identify at least one supportive staff member [70]. Even though a greater sense of safety is achieved in the presence of supportive individuals, LGBTQ youth continue to rank athletic coaches and physical education teachers the lowest (20.8%) among high school personnel of whom students would be comfortable talking with one-on-one about LGBTQ issues, even below principals and resource officers. Furthermore, more than one fifth (22.0%) of LGBTQ students indicated that the influence of school officials and coaches had discouraged or prevented their peers from sport participation, while one tenth (10.8%) of students indicated that it happened to them personally [70].

Being a role model is one of the most powerful actions that sports medicine professionals can take whether in the clinic, on the sidelines, out in the community, or in the athletic training room [15]. If a student-athlete perceives their sports medicine team as accepting and open-minded about diverse sexual orientations, they will likely be more comfortable in receiving care and opt to seek care when necessary. However, sports medicine personnel must not ignore their personal assumptions and biases surrounding heteronormativity and homonegativity, particularly in how their personal biases affect interactions with LGB student-athletes and their ability to provide high-quality care [15]. By educating oneself on the unique needs of the LGB athlete and appropriately addressing one’s own assumptions, providers will continue to promote a change in heterosexist attitudes and create a more inclusive environment to extend beyond the sports medicine profession.

Sports medicine professionals have the tremendous potential to influence the lifelong relationship and attitude of the LGB population towards seeking preventative and routine medical care. Communication difficulty, prejudicial conduct of healthcare professionals, perceived need for holistic care beyond sexual issues, and fear of sexual orientation disclosure are just a few of the historical factors providing barriers to comprehensive healthcare among LGB patients [71, 72]. A recent systematic review highlights heteronormative attitudes imposed by healthcare professionals, as well as fear of rejection and breach of confidentiality among LGB patients, leading to unmet healthcare needs and patient dissatisfaction [71]. This further promoted decreased utilization of routine health services, increased use of emergency services for acute care, and risk of self-medication. Additionally, delayed access to care was related to higher rates of chronic medical conditions in the LGB population such as sexually transmitted diseases (STDs), HIV/AIDS, untreated mental health conditions, substance abuse disorders, and detectable cancers. If an LGB individual has a positive experience with the sports medicine team as an adolescent and early adult, this may have the potential to offset previous alienating healthcare experiences and encourage appropriate medical care in the future.

## Recommendations for Action

As LGB athletes become more visible not only in the intercollegiate setting, but also in the professional and Olympic arenas, policies and practices regarding eligibility and inclusivity will unquestionably continue to be developed and discussed. Despite the recent increased attention and focus on inclusive and welcoming athletic environments for LGB student-athletes promoted by the NCAA, reported findings suggest that these individuals continue to remain marginalized due to their sexual orientation. Athletic participation should be a protective factor and provide an additional layer of support and belonging for LGB student-athletes, but this is untrue in the current Western culture of sport. Not only are LGB collegiate student-athletes significantly burdened by perceived homophobic attitudes and fear of “outing” their non-conforming sexual identity in the heterosexist culture of sport, but their participation in intercollegiate sport is also challenged by barriers of increased rates of non-accidental violence, homonegative discrimination, mental health disorders, substance use disorders, and physical inactivity. If marginalized student-athletes are relieved of the worry and anxiety associated with hiding aspects of their sexual orientation, they will be better able to focus on team and individual athletic and academic goals.

As it is evident that a greater sense of safety and security is achieved with identification of a supportive staff, this offers an opportunity for the sports medicine team to advocate for an inclusive environment formally and visibly in order to develop trust among LGB student-athletes inside the athletic training room. Insensitivity and silence in response to the reported negative health outcomes perpetuated by a non-inclusive environment poses a direct threat to the overall athletic performance, team chemistry, response to injury treatment, and perceived quality of medical care received. These negative outcomes can further be potentiated by delayed access to appropriate medical care and failure of implementation of sport safety rules in training and competition environments. Awareness of inequity and non-inclusive climates faced by LGB student-athletes ultimately must translate into action by implementation of inclusive practices and consequences for discriminatory and homophobic behaviors. Best healthcare practices for providers should include appropriate and inclusive language, visible and supportive presence for LGB student-athletes, self-reflection to monitor one’s own beliefs and biases, inclusive policies at the institutional level, and increased awareness and understanding of LGB issues and concerns [6, 12, 13].While it is realized that not every athletic department will have the infrastructure and resources to provide a multidisciplinary care team of mental health professionals, sport psychology consultants, sports medicine staff, and counselors available for student-athletes onsite, sports medicine providers still have a responsibility to prioritize equal access for all student-athletes to interdisciplinary healthcare teams and specialized professionals. By promoting the visibility of a more inclusive environment for LGB collegiate student-athletes and addressing the holistic care of these athletes from a multidisciplinary approach, health benefits from organized team sport and physical activity will reach a larger audience that includes marginalized LGB youth.

## Future Direction for Scholarship

Sports medicine professionals should be leaders in studying the barriers that LGB student-athletes face, not only in athletic competition and social acceptance, but also in access to comprehensive healthcare. Multidisciplinary research is needed to better understand the wellness needs of LGB student-athletes. Qualitative and quantitative studies of LGB individuals who previously participated in collegiate athletics would be beneficial to examine individual athletic performance, attitudes and perceived bias among sports medicine staff, response to medical treatment, return to play decisions, and quality of care received from the sports medicine team in relation to heterosexual peers. To better understand the psychosocial aspect of athletic participation as a potential benefit to character development and well-being, quantitative data comparing academic performance, graduation rates, transfer percentages, and drop-out rates among LGB athletes and heterosexual peers would also be a significant contribution to the literature. Further qualitative assessment of the perception of athletics to personal and social identity as well as adjustment to life after sport among LGB and heterosexual athletes would also be worthy of exploration.

Additionally, it is imperative that future work develops educational and athletic agendas in parallel, as these two disciples are so closely intertwined at the collegiate level. As policy continues to change, future research must continue to assess the translation of publicly and openly LGB administrators, coaches, educators, and athletic training staff to the perception of an accepting and safe environment among LGB student-athletes. In order to train culturally and socially sensitive medical providers, pre-professional education programs should be designed across all levels of medical professionals training to incorporate a dedicated curriculum on the specific needs of the LGB population in order to reduce homophobic attitudes and promote awareness of unique obstacles among this population.

This study is limited by a lack of a systematic review methodology to include all pertinent articles from the current literature. However, the narrative review methodology allowed us to be broader in scope and more exploratory in our approach to the sizable current gaps in the literature with regard to this subject. Additionally, some of our conclusions were based on careful extrapolation from studies of either non-athlete LGB students or heterosexual student-athlete peers due to the lack of current literature addressing the realm of our discussion. However, this limitation will only be overcome by future qualitative and quantitative studies comparing LGB student-athletes to their heterosexual peers as discussed above.

## Conclusions

To complement the NCAA mission of equal opportunity throughout all policies and practices and to enhance and diversify the learning environment for all participating student-athletes, emphasis needs to focus on more inclusive practices to engage LGB student-athletes throughout intercollegiate sports. As first-line providers for all student-athletes, sports medicine physicians and athletic training personnel have the opportunity and professional responsibility to advocate for the holistic health of every student-athlete to specifically include those marginalized as sexual minorities. Barriers and biases faced by LGB student-athletes can be addressed by sports medicine personnel through identifying resources for multidisciplinary care, monitoring personal beliefs and biases, educating providers on the unique prejudices and discrimination against LGB student-athletes, providing a visible support system of advocacy, and protecting the confidentiality of student-athlete sexual orientation. Awareness of the barriers and biases previously discussed should encourage all sports medicine professionals to dismantle the current heteronormative culture of sport at an individual and institutional level by advocating for an environment in which all student-athletes can equally and openly participate in athletic competition.

## Abbreviations

LGB: Lesbian, gay, and bisexual
LGBT: Lesbian, gay, bisexual, and transgender
LGBTQ: Lesbian, gay, bisexual, transgender, and queer
NAIA: National Association of Intercollegiate Athletics
NBA: National Basketball Association
NCAA: National Collegiate Athletic Association
NFL: National Football League
STDs: Sexually transmitted diseases
WNBA: Women’s National Basketball Association
